# Supplementary material for: The Impact of Urbanization on Taxonomic Diversity and Functional Similarity among Butterfly Communities in Waterfront Green Spaces
Source: Insects. 2023 Oct 31;14(11):851. doi: 10.3390/insects14110851 (PMC10671622; doi:10.3390/insects14110851)
Supplement: Supplementary file 1 [file insects-14-00851-s001.zip › insects-2625911-supplementary.pdf]

Table S1: Butterfly species introduction, space, food habits, and number of individuals.

No.	Latin name	Family	Genera	Space	Diet	Individuals
1	*Pseudozizeeria maha (Kollar,1844)*	Lycaenidae	Pseudozizeeria	L	P	327
2	*Catopsilia pomona (Fabricius, 1775)*	Pieridae	Catopsilia	U	P	295
3	*Pieris rapae (Linnaeus,1758)*	Pieridae	Pieris	M	P	167
4	*Argynnis hyperbius (Linnaeus,1763)*	Nymphalidae	Argynnis Fabricius	M	O	114
5	*Eurema hecabe (Linnaeus,1758)*	Lycaenidae	Arhopala	L	P	74
6	*Chilades pandava (Horsfield,1829)*	Pieridae	Eurema	M	P	51
7	*Pieris canidia (Linnaeus,1768)*	Pieridae	Pieris	M	P	21
8	*Graphium sarpedon (Linnaeus,1758)*	Papilionidae	Graphium	U	O	20
9	*Taraka hamada (Druce,1875)*	Lycaenidae	Taraka	L	C	20
10	*Papilio polytes (Linnaeus,1758)*	Papilionidae	PapilioLinnaeus	U	O	16
11	*Papilio xuthus (Linnaeus,1767)*	Papilionidae	Papilio	U	O	10
12	*Elymnias hypermnestra (Linnaeus,1763)*	Nymphalidae	Elymnias	M	O	9
13	*Colias erate (Esper,1805)*	Pieridae	Colias	M	P	8
14	*Polygonia c-aureum (Linnaeus,1758)*	Nymphalidae	Polygonia	M	O	5
15	*Papilio helenus (Linnaeus,1758)*	Papilionidae	Papilio	U	O	3
16	*Hestina assimilis (Linnaeus,1758)*	Nymphalidae	Hestina	U	SP	3
17	*Vanessa cardui (Linnaeus,1758)*	Nymphalidae	Vanessa	M	P	3
18	*Parnara ganga (Evans,1937)*	Hesperiidae	Parnara Moore	M	O	3
19	*Papilio memnon (Linnaeus,1758)*	Papilionidae	Papilio	U	O	2
20	*Junonia almana (Linnaeus,1758)*	Nymphalidae	Junonia	M	P	2
21	*Ypthima motschulskyi (Bremer & Grey, 1853)*	Satyridae	Ypthima	M	SP	2
22	*Graphium doson (Felder & Felder, 1864)*	Papilionidae	Graphium	U	O	2
23	*Vanessa indica (Herbst, 1794)*	Nymphalidae	Vanessa	M	O	1
24	*Neptis hylas (Linnaeus, 1758)*	Nymphalidae	Neptis	M	P	1
25	*Papilio paris (Linnaeus, 1758)*	Papilionidae	Papilio	U	P	1
26	*Kallima inachus (Boisduval, 1836)*	Nymphalidae	Kallima	U	S	1
27	*Mycalesis gotama (Moore, 1857)*	Satyridae	Mycalesis	M	S	1
28	*Menelaides protenor (Cramer,1775)*	Papilionidae	Papilio	U	O	1

Note: U=upper activity space; M=middle activity space, L=lower activity space; SP=Plant-feeding monophagous, O=Plant-feeding oligophagous; P=Plant-feeding polyphagous; C=Carnivorous; S=Scavenging.

Table S2: The butterfly community indices of urban green spaces were measured across four levels of urbanization gradients, encompassing indices of Butterfly α-diversity, butterfly richness, butterfly abundance, and the butterfly Chao1 index.

Urbanization Gradient Type	Functional Group	Butterflies Group Variable	Parameter			
			Butterflies α-diversity	Butterflies Richness	Butterflies Abundance	Butterflies Chao1
City center areas	All over	All butterflies	1.173	8	353	9
	Diet group	Plant-feeding monophagous	——	——	——	——
		Plant-feeding oligophagous	1.367	5	11	8
		Plant-feeding polyphagous	1.024	3	342	3
		Carnivorous butterfly	——	——	——	——
		Scavenging butterfly	——	——	——	——
	Vertical activity space group	Upper space activities	0.254	3	69	3
		Middle space activities	0.284	4	112	5
		Lower space activities	0.000	1	172	1
Urban areas	All	All butterflies	1.737	14	374	14.5
	Diet group	Plant-feeding monophagous	——	——	——	——
		Plant-feeding oligophagous	1.036	5	51	5
		Plant-feeding polyphagous	1.251	8	307	9
		Carnivorous butterfly	——	——	——	——
		Scavenging butterfly	——	——	——	——
	Vertical activity space group	Upper space activities	0.342	4	160	4
		Middle space activities	1.387	7	87	8
		Lower space activities	0.584	3	127	3
Sub-urban areas	All over	All butterflies	2.037	16	267	19
	Diet group	Plant-feeding monophagous	0.000	1	3	1
		Plant-feeding oligophagous	0.881	4	54	4
		Plant-feeding polyphagous	1.602	9	208	9.5
		Carnivorous butterfly	0.000	1	1	1
		Scavenging butterfly	0.000	1	1	1
	Vertical activity space group	Upper space activities	0.811	7	101	8
		Middle space activities	1.457	6	127	6
		Lower space activities	0.238	3	39	4
	All over	All butterflies	1.975	17	169	17.6

Ex-urban areas	Diet group	Plant-feeding monophagous	0.000	1	2	1
		Plant-feeding oligophagous	1.439	8	70	8
		Plant-feeding polyphagous	0.965	6	93	6
		Carnivorous butterfly	0.000	1	3	1
		Scavenging butterfly	0.000	1	1	1
	Vertical activity space group	Upper space activities	1.410	6	24	6.333
		Middle space activities	1.322	8	62	8
		Lower space activities	0.602	3	83	3
All butterflies	All over	All butterflies	——	28	1163	——
	Diet group	Plant-feeding monophagous	——	2	5	——
		Plant-feeding oligophagous	——	12	186	——
		Plant-feeding polyphagous	——	11	950	——
		Carnivorous butterfly	——	1	20	——
		Scavenging butterfly	——	2	2	——
	Vertical activity space group	Upper space activities	——	11	354	——
		Middle space activities	——	14	388	——
		Lower space activities	——	3	421	——

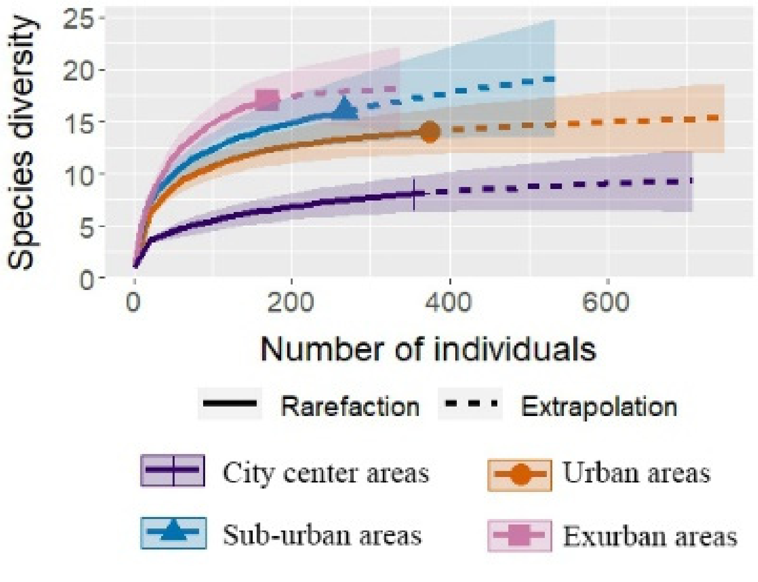

Figure S1: The rarefaction curve based on the number of individuals of species and the extrapolation curve of butterfly diversity in waterfront green spaces with different urbanization types. When the rarefaction curve flattens, the observed species diversity will stabilize gradually, and more individuals will only produce fewer new species.

Table S3: The table displays linear regression models (LRM)that illustrate the relationship between Shannon and species abundance of butterfly functional groups in urban green space under various types of urbanization. The

abbreviations used in the table have the following meanings: POD indicates Shannon of plant-feeding oligophagous butterflies, POP indicates Shannon of plant-feeding polyphagous butterflies, UAS indicates Shannon of upper activity space, MAS indicates Shannon of middle activity space, LAS indicates Shannon of lower activity space, and SA indicates species abundance.

Node	Butterfly Groups Type	Type of urbanization	(LRM)Model	F	R^2	Sig.
1	Plant-feeding oligophagous	City center areas	POD=0.137SA+0.437	0.7172	0.082	0.422
2		Urban areas	POD=0.035 SA +0.691	7.476	0.418	0.026*
3		Suburban areas	POD=0.025 SA +0.136	3.709	0.317	0.09
4		Exurban areas	POD=0.039 SA +0.268	7.975	0.499	0.022*
5	Plant-feeding polyphagous	City center areas	POP=0.170SA-0.044	13.81	0.633	0.006*
6		Urban areas	POP=0.005SA+0.706	0.892	0.100	0.372
7		Suburban areas	POP=0.017SA+0.506	2.572	0.243	0.147
8		Exurban areas	POP=0.290SA-0.110	0.034	0.004	0.859
9	Upper space activities	City center areas	UAS=-0.003SA+0.164	0.066	0.008	0.804
10		Urban areas	UAS=-0.011SA+0.573	3.193	0.285	0.112
11		Suburban areas	UAS=-0.003SA+0.515	0.024	0.003	0.881
12		Exurban areas	UAS=0.128SA-0.002	7.243	0.475	0.027*
13	Middle space activities	City center areas	MAS=0.002SA+0.641	0.421	0.050	0.535
14		Urban areas	MAS=0.058SA+0.163	8.248	0.508	0.021*
15		Suburban areas	MAS=0.030SA+0.177	20.21	0.716	0.002*
16		Exurban areas	MAS=0.02SA+0.282	11.180	0.129	0.309
17	Lower space activities	City center areas	LAS=0.000SA+0.000	0.000	0.000	0.000
18		Urban areas	LAS=0.008SA-0.044	32.25	0.815	0.001*
19		Suburban areas	LAS=0.008SA+0.059	0.321	0.039	0.587
20		Exurban areas	LAS=0.239SA-0.048	0.703	0.081	0.426

Table S4: We identified the species with the strongest correlation, determined their indicator value (IV), and evaluated the statistical significance of the correlation using a P-value threshold of <0.05*.

Node	Butterflies Indicator	Type of Urban Gradient	IndVal (IV)	Frequency	P-Value
1	*Pieris rapae*	City center areas	0.377	21	0.019*
2	*Catopsilia pomona*	Urban areas	0.502	28	0.007*
3	*Eurema hecabe*	Sub-urban areas	0.563	12	0.004*
4	*Chilades pandava*	Exurban areas	0.714	10	0.001*
5	*Papilio xuthus*	Exurban areas	0.300	3	0.049*
